# Supplementary material for: Multi‐dataset identification of innovative feature genes and molecular mechanisms in keratoconus
Source: J Cell Mol Med. 2024 Sep 19;28(18):e70079. doi: 10.1111/jcmm.70079 (PMC11412914; doi:10.1111/jcmm.70079)
Supplement: Supplementary file 2 — Material S2. [file JCMM-28-e70079-s002.zip › 7.GSEA analysis/3.DBNDD1/KEGG_THYROID_CANCER.html]

Details for gene set KEGG\_THYROID\_CANCER[GSEA]

|  || Dataset | DBNDD1.group.cls#h\_versus\_l |
| Phenotype | group.cls#h\_versus\_l |
| Upregulated in class | l |
| GeneSet | KEGG\_THYROID\_CANCER |
| Enrichment Score (ES) | -0.44369212 |
| Normalized Enrichment Score (NES) | -1.5780963 |
| Nominal p-value | 0.028455285 |
| FDR q-value | 0.56209546 |
| FWER p-Value | 0.646 |
Table: GSEA Results Summary

  

Fig 1: Enrichment plot: KEGG\_THYROID\_CANCER      
 Profile of the Running ES Score & Positions of GeneSet Members on the Rank Ordered List

  

| SYMBOL | TITLE | RANK IN GENE LIST | RANK METRIC SCORE | RUNNING ES | CORE ENRICHMENT || 1 | CCND1 | na | 9676 | 0.076 | -0.2454 | No |
| 2 | MAPK3 | na | 9865 | 0.073 | -0.2185 | No |
| 3 | NTRK1 | na | 10107 | 0.070 | -0.1944 | No |
| 4 | MAP2K2 | na | 10931 | 0.059 | -0.1921 | No |
| 5 | RET | na | 11988 | 0.047 | -0.2018 | No |
| 6 | RXRA | na | 12017 | 0.047 | -0.1818 | No |
| 7 | TP53 | na | 13962 | 0.030 | -0.2245 | No |
| 8 | KRAS | na | 14127 | 0.029 | -0.2164 | No |
| 9 | BRAF | na | 15471 | 0.019 | -0.2465 | No |
| 10 | NCOA4 | na | 16364 | 0.013 | -0.2663 | No |
| 11 | CTNNB1 | na | 16378 | 0.013 | -0.2608 | No |
| 12 | CDH1 | na | 18305 | 0.001 | -0.3160 | No |
| 13 | HRAS | na | 18984 | -0.004 | -0.3336 | No |
| 14 | TCF7L2 | na | 20073 | -0.014 | -0.3589 | No |
| 15 | TFG | na | 20631 | -0.019 | -0.3668 | No |
| 16 | RXRB | na | 21455 | -0.026 | -0.3789 | No |
| 17 | CCDC6 | na | 21772 | -0.030 | -0.3749 | No |
| 18 | MYC | na | 22660 | -0.039 | -0.3832 | No |
| 19 | MAPK1 | na | 22705 | -0.040 | -0.3669 | No |
| 20 | TPM3 | na | 23026 | -0.043 | -0.3569 | No |
| 21 | TCF7 | na | 23571 | -0.051 | -0.3503 | No |
| 22 | TCF7L1 | na | 26813 | -0.104 | -0.3979 | Yes |
| 23 | NRAS | na | 27014 | -0.108 | -0.3559 | Yes |
| 24 | RXRG | na | 28564 | -0.145 | -0.3365 | Yes |
| 25 | TPR | na | 28794 | -0.151 | -0.2763 | Yes |
| 26 | MAP2K1 | na | 29246 | -0.164 | -0.2168 | Yes |
| 27 | PAX8 | na | 30919 | -0.218 | -0.1687 | Yes |
| 28 | PPARG | na | 32783 | -0.313 | -0.0842 | Yes |
| 29 | LEF1 | na | 32857 | -0.317 | 0.0539 | Yes |
Table: GSEA details [plain text format]

  

Fig 2: KEGG\_THYROID\_CANCER      
 Blue-Pink O' Gram in the Space of the Analyzed GeneSet

  

Fig 3: KEGG\_THYROID\_CANCER: Random ES distribution      
 Gene set null distribution of ES for **KEGG\_THYROID\_CANCER**
